# Supplementary figures and images for: Haplotype of the astrocytic water channel AQP4 is associated with slow wave energy regulation in human NREM sleep
Source: PLoS Biol. 2020 May 5;18(5):e3000623. doi: 10.1371/journal.pbio.3000623 (PMC7199924; doi:10.1371/journal.pbio.3000623)

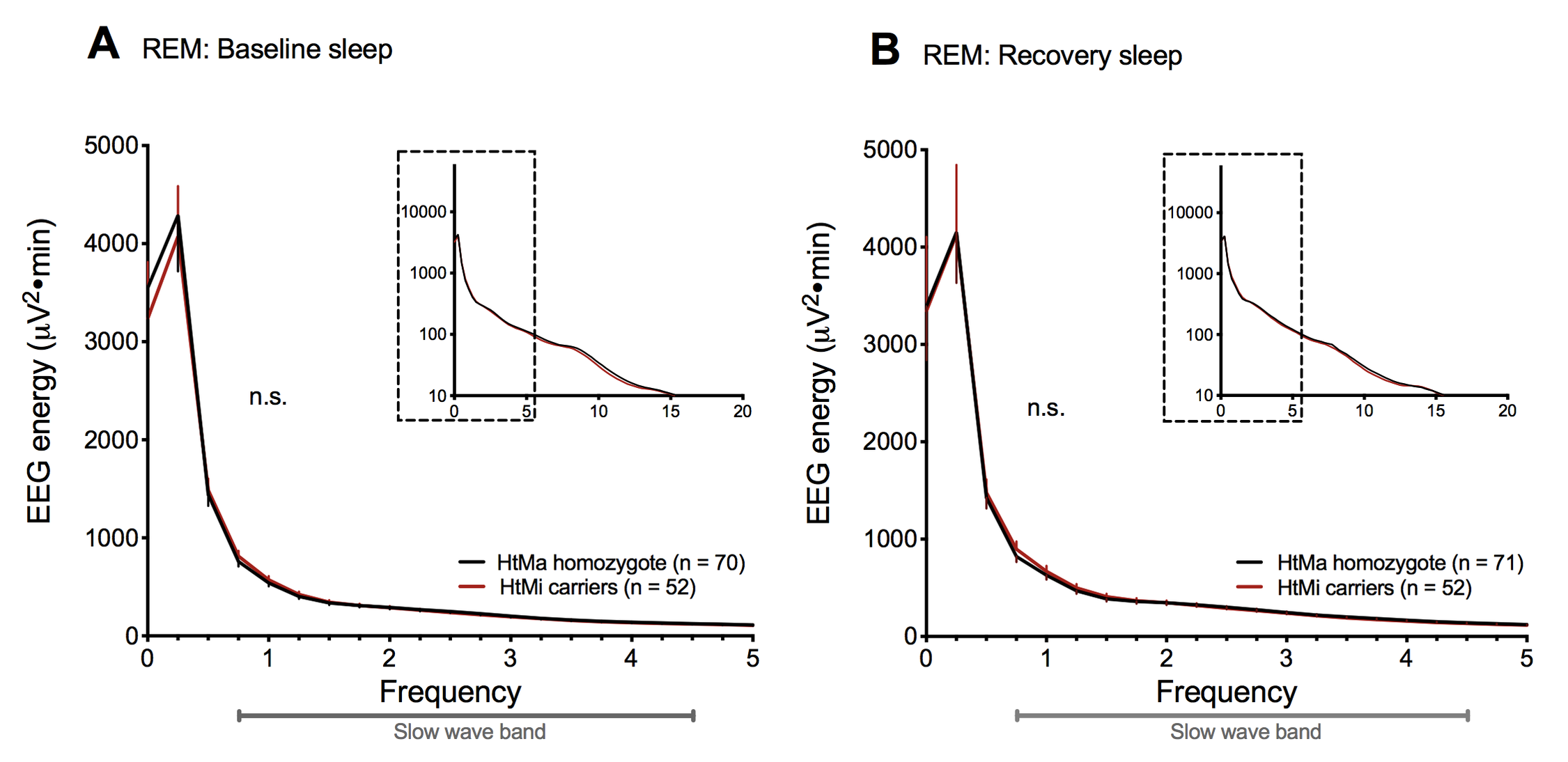

Supplement: S1 Fig — REM energy analysis for AQP4 HtMa homozygous (black lines) and HtMi carriers (red lines) across baseline (A) nor recovery (B) nights. No significant modulation by the AQP4 haplotype was observed (“haplotype”: F1,21 = 0.07; P > 0.79), confirming that the sleep EEG modulations are selective to the NREM slow wave range (S5 Data). Plots represent means; error bars represent SEM. (Insert) Full NREM sleep spectra for 0 to 20 Hz on log10 scale. AQP4, aquaporin 4; EEG, electroencephalographic; HtMa, Major allele of haplotype; HtMi, Minor allele of haplotype; NREM, non–rapid eye movement; REM, rapid eye movement. (TIF) [file pbio.3000623.s001.tif]

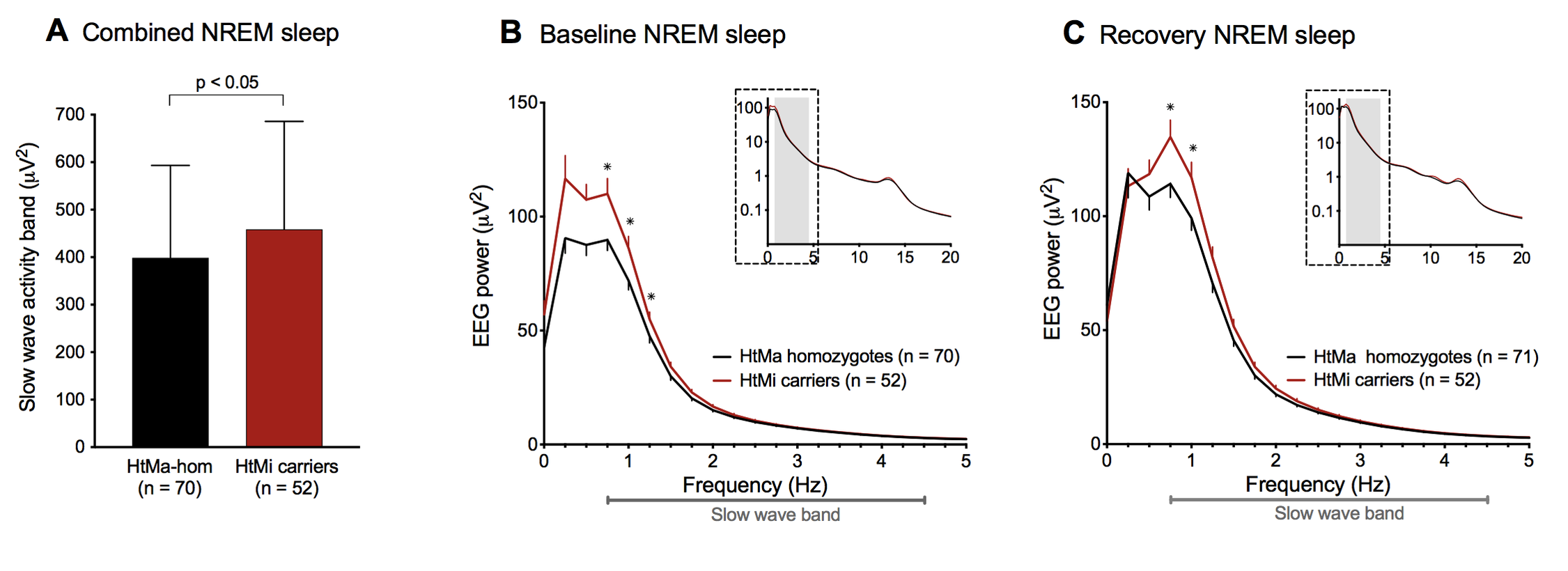

Supplement: S2 Fig — Comparison of EEG spectral power across baseline and recovery nights in the slow wave band (0.75–4.5 Hz) within the AQP4-haplotype variants HtMa homozygotes (black) and HtMi carriers (red). The AQP4 HtMi carriers had higher spectral power than the HtMa homozygotes (A; “genotype”: F1,121 = 4.2; P < 0.05). The effect was similar in baseline (B) and recovery sleep (C) conditions and confined to the 0.75 to 1.25 Hz band. Inserts in panels B and C represent full NREM sleep spectra for 0 to 20 Hz on log10 scale. Gray shading indicating the slow wave band (S2 Data). Data represents means ± SEM. By-bin unpaired two-tailed t tests; *P < 0.05 (only performed in the slow wave range). AQP4, aquaporin 4; EEG, electroencephalographic; HtMa, Major allele of haplotype; HtMi, Minor allele of haplotype; NREM, non–rapid eye movement. (TIF) [file pbio.3000623.s002.tif]
