# Supplementary material for: Haplotype of the astrocytic water channel AQP4 is associated with slow wave energy regulation in human NREM sleep
Source: PLoS Biol. 2020 May 5;18(5):e3000623. doi: 10.1371/journal.pbio.3000623 (PMC7199924; doi:10.1371/journal.pbio.3000623)
Supplement: S1 Table — Demographic characteristics of the 123 healthy adult volunteers, who participated in one of six 40-hour sleep deprivation studies from the Zürich sleep lab where AQP4 HtMa homozygotes and HtMi allele carriers were genotyped. No demographic differences between the 2 AQP4-haplotype groups was observed, nor was there a difference in haplotype distribution among the 6 studies (Fishers exact t test, p > 0.21). Given the low number of females in the 6 included studies, a potential interaction between AQP4 haplotype and gender could not be addressed in the current paper. German versions and validated German translations of questionnaires were used to assess lifestyle and personality traits. Questionnaires included ESS [42] and STAI [43]. Caffeine consumption was estimated based on average caffeine contents per serving (coffee: 100 mg, tea: 30 mg, cola drink: 40 mg [2 dL], energy drink: 80 mg [2 dL], chocolate: 50 mg [100 g]). The APOE genotype, known to modulate Alzheimer disease progression, was evenly distributed among the AQP4 haplotype groups. P values are calculated from students two-tailed t tests or Fisher’s exact test where appropriate. APOE, Apolipoprotein E; AQP4, aquaporin 4; ESS, Epworth Sleepiness Scale; HtMa, Major allele of haplotype; HtMi, Minor allele of haplotype; STAI, State-Trait Anxiety Inventory. (DOCX) [file pbio.3000623.s003.docx]

| **S1 Table**  **Demographics** | | | | |
| --- | --- | --- | --- | --- |
|  | **HtMa homozygotes** | **HtMi carriers** | **P-value** |  |
| Sample size (n_total_ = 123) | 71 | 52 |  |  |
| Age (years) | 24.1 ± 2.8 | 23.9 ± 3.0 | 0.69 |  |
| BMI (kg/cm^2^) | 22.4 ± 1.6 | 22.6 ± 2.1 | 0.40 |  |
| Gender (% females) | 12,7 | 3.8 | 0.12 |  |
| Reported habitual sleep duration (h) | 7.3 ± 0.7 | 7.4 ± 0.7 | 0.46 |  |
| Trait anxiety (STAI) | 33.25 ± 7.1 | 35.1 ± 8.5 | 0.19 |  |
| Sleepiness (ESS) | 6.6 ± 3.0 | 6.9 ± 2.9 | 0.61 |  |
| Smoking (% yes) | 2.8 | 1.9 | 1 |  |
| Caffeine consumption (mg/day) | 108.1 ± 100.3 | 129.9 ± 118.9 | 0.27 |  |
| Alcohol consumption (drinks/week) | 3.2 ± 2.7 | 2.8 ± 2.6 | 0.43 |  |
| APOE genotype |  |  |  |  |
| ε2 carrier | 10 | 4 |  |  |
| ε3 carrier | 42 | 36 |  |  |
| ε4 carrier | 16 | 11 | 0.47 |  |
