# Supplementary material for: Haplotype of the astrocytic water channel AQP4 is associated with slow wave energy regulation in human NREM sleep
Source: PLoS Biol. 2020 May 5;18(5):e3000623. doi: 10.1371/journal.pbio.3000623 (PMC7199924; doi:10.1371/journal.pbio.3000623)
Supplement: S2 Table — Alleles: presented as [major allele/minor allele]. Position: Position of SNP in gene. Location: Location of single nucleotid polymorphism on chromosome 18 in the genome. Assay ID: Thermofisher Taqman® SNP genotype assay ID nr. MAFpredicted: MAF predicted by dbSNP analysis tool in a CEU and TSI population (to approximate the Swiss population). MAFstudy: MAF in the entire genotyped study population (n = 134), including 2 rare genotypes and 9 elderly subjects excluded from analysis. dbSNP, The Single Nucleotide Polymorphism Database; CEU, Utah Residents from North and West Europe; MAF, minor allele frequency; SNP, single nucleotide polymorphism; TSI, Toscani in Italy; (DOCX) [file pbio.3000623.s004.docx]

|  | **S2 Table.** **Investigated single nucleotide polymorphisms (SNPs)** | | | | | |  |  |
| --- | --- | --- | --- | --- | --- | --- | --- | --- |
|  | | Alleles | position | Location in genome | Assay ID | MAF_predicted_ | | MAF_study_ |
| rs335931 | | A / G | Intron 4-5 | Chr.18: 26859108 | C__11630001_10 | 0.202 | | 0.243 |
| rs335929 | | A / C | 3UTR | Chr.18: 26855623 | C__1303566_10 . | 0.202 | | 0.235 |
| rs16942851 | | T / G | Downstream  of 3UTR | Chr.18: 26851725 | C__33451186_20 | 0.202 | | 0.239 |
