# Supplementary material for: Haplotype of the astrocytic water channel AQP4 is associated with slow wave energy regulation in human NREM sleep
Source: PLoS Biol. 2020 May 5;18(5):e3000623. doi: 10.1371/journal.pbio.3000623 (PMC7199924; doi:10.1371/journal.pbio.3000623)
Supplement: S3 Table — Data on the visually scored sleep variables and their modulation by sleep deprivation and the AQP4 haplotype. Values represent mean ± SEM in baseline and recovery nights for the 2 haplotype groups. Analysis of the recovery nights were restricted to 480 minutes. Sleep efficiency: percentage of total sleep time per 480 min. Stages N1–N3: NREM sleep stages (N3 refers to slow wave sleep). Sleep latency: time from lights-out to the first occurrence of N2 sleep. REM sleep latency: time from sleep onset to the first occurrence of REM sleep. F- and P values: two-way mixed-model ANOVA with factors “genotype” (HtMa homozygotes, HtMi carriers), “condition” (baseline, recovery), and their interaction. AQP4, aquaporin 4; HtMa, Major allele of haplotype; HtMi, Minor allele of haplotype; MT, movement time; NREM, non–rapid eye movement; REM, rapid eye movement; TIB, time in bed; TST, total sleep time; WASO, wakefulness after sleep onset. (DOCX) [file pbio.3000623.s005.docx]

**S3 Table. Visually scored sleep variables**

|  | **HtMa-hom (n = 71)** | | **HtMi carriers (n = 52)** | | **‘Genotype’** | | **‘Condition’** | | | **‘Genotype’ x ‘Condition’** | |
| --- | --- | --- | --- | --- | --- | --- | --- | --- | --- | --- | --- |
|  | **Baseline** | **Recovery** | **Baseline** | **Recovery** | **F_1,121_** | **P** | **F_1,121_** | | **P** | **F_1,121_** | **P** |
| **Sleep efficiency (%)** | 94.0 ± 0.4 | 97.5 ± 0.2 | 94.1 ± 0.5 | 97.8 ± 0.2 | 0.3 | 0.59 | *131.0* | | *<0.0001* | 0.1 | 0.75 |
| **Sleep latency (min)** | 14.7 ± 1.3 | 3.5 ± 0.3 | 14.8 ± 1.6 | 3.4 ± 0.6 | 0 | 0.97 | *119.4* | | *<0.0001* | 0.01 | 0.92 |
| **REM sleep latency (min)** | 76.0 ± 3.5 | 81.3 ± 4.9 | 68.5 ± 2.9 | 71.0 ± 3.9 | *4.3* | *0.04* | | 1.1 | 0.31 | 0.2 | 0.71 |
| **Stage N1 (min)** | 30.9 ± 2.0 | 14.8 ± 1.2 | 26.9 ± 1.9 | 12.1 ± 1.5 | 2.3 | 0.13 | *218.4* | | *<0.0001* | 0.4 | 0.55 |
| **Stage N2 (min)** | 215.7 ± 4.1 | 201.6 ± 4.8 | 219.9 ± 5.2 | 207.3 ± 5.6 | 0.6 | 0.45 | *25.9* | | *<0.0001* | 0.08 | 0.77 |
| **Stage N3 (min)** | 95.3 ± 4.1 | 149.2 ± 4.9 | 101.4 ± 5.5 | 151.2 ± 5.7 | 0.3 | 0.56 | *687.1* | | *<0.0001* | 1.1 | 0.29 |
| **NREM sleep (min)** | 316.4 ± 3.5 | 352.8 ± 3.1 | 323.6 ± 3.2 | 359.2 ± 3.8 | 2.5 | 0.11 | *204.5* | | *<0.0001* | 0.02 | 0.89 |
| **REM sleep (min)** | 109.0 ± 2.8 | 101.9 ± 3.1 | 103.0 ± 2.7 | 99.0 ± 3.6 | 1.4 | 0.25 | *6.27* | | *0.014* | 0.5 | 0.49 |
| **TST (min)** | 450.9 ± 2.0 | 467.5 ± 1.0 | 451.3 ± 2.3 | 469.6 ± 0.8 | 0.5 | 0.50 | *126.5* | | *<0.0001* | 0.3 | 0.59 |
| **MT (min)** | 6.7 ± 0.6 | 6.7 ± 0.6 | 6.5 ± 0.6 | 5.8 ± 0.6 | 0.5 | 0.49 | 1.7 | | 0.20 | 1.4 | 0.25 |
| **WASO (min)** | 7.0 ± 1.2 | 1.1 ± 0.3 | 5.9 ± 1.5 | 1.2 ± 0.4 | 0.3 | 0.61 | *35.0* | | *<0.0001* | 0.4 | 0.52 |
| **TIB (min)** | 479.7 ± 0 | 479.4 ± 0.6 | 479.6 ± 0.1 | 480 ± 0 | 0.5 | 0.47 | 0.01 | | 0.92 | 1.0 | 0.32 |
|  | | | | | | | | | | | |
