# Supplementary material for: Haplotype of the astrocytic water channel AQP4 is associated with slow wave energy regulation in human NREM sleep
Source: PLoS Biol. 2020 May 5;18(5):e3000623. doi: 10.1371/journal.pbio.3000623 (PMC7199924; doi:10.1371/journal.pbio.3000623)
Supplement: Attachment — Submitted filename: ResponsetoReviewers_AQP4_sleep_19032020.docx [file pbio.3000623.s011.docx]

\# PBIOLOGY-D-19-03561R1

**Haplotype of the astrocytic water channel AQP4 is associated with slow wave energy regulation in human NREM sleep**

*Sara Marie Ulv Larsen ^1^, Hans-Peter Landolt^2,3,4,8^, Wolfgang Berger^3,4,5^, Maiken Nedergaard^6^, Gitte Moos Knudsen^1,7^, Sebastian Camillo Holst^1,8^**

^1^ Neurobiology Research Unit, Rigshospitalet, Copenhagen, Denmark

^2^ Institute of Pharmacology & Toxicology, University of Zurich, Zurich, Switzerland

^3^Zurich Center for Integrative Human Physiology, University of Zurich, 8057 Zurich, Switzerland.

^4^Neuroscience Center Zurich, University and ETH Zurich, 8057 Zurich, Switzerland.

^5^Institute of Medical Molecular Genetics, University of Zurich, Schlieren, Switzerland

^6^Center for Translational Neuromedicine, University of Copenhagen, Copenhagen, Denmark

^7^ Faculty of Health and Medical Sciences, University of Copenhagen, Copenhagen, Denmark

^8^ Sleep & Health Zurich, University Center of Competence, Zurich, Switzerland

*** E-mail for correspondence:** holst@nru.dk

**Responses to the Referee Report**

We thank the three Reviewers, the Editorial team and the Senior Editor for their careful and detailed evaluation of our submission to *PLoS Biology*. In our response, we address all comments made and issues raised by them and indicate all changes in the revised version of the manuscript. We are persuaded that the revisions based on the Reviewers’ thoughtful and constructive criticism improved the paper.

**Reviewers’ general comments:**

**Reviewer #1:**

*“This is an interesting and well-written paper that shows that AQP4 haplotype predicts NREM slow wave energy and response to sleep deprivation. The paper is clear and the analyses are well conducted. I have some comments on the analysis and interpretation.”*

**Authors’ response:** We thank the Reviewer for the positive general evaluation of our work and will address the comments in the following.

1*. “- The authors state that there was no difference in other frequency bands, with "data not shown". Since the full spectrum is in the fig. 2 panel inset, could this be replaced with a statement of (p>0.05) instead, or of some other metric of how they determined lack of a difference? This seems like an important point to back up with data, and it appears to me that the data are present.”*

**Authors’ response:** We agree this is a relevant question. Because we selected to use a fixed sequence procedure (see Statistical analyses section of the Methods p. 14), we stopped post-hoc testing after observing a non-significant modulation in the spindle range (P > 0.77), which was our second band of choice in this hypothesis driven analysis. For the same reason, the 0-20 Hz spectrum (fig. 2 inset) does not include bin-wise statistical testing. Such tests were only performed in the slow wave band which revealed an overall significant modulation by the *AQP4*-haplotype.

However, based on the reviewers’ question, we now performed post-hoc analysis of the remaining EEG-energy bands. In line with our initial hypothesis, we didn’t find any significant differences in NREM energy between the two haplotypes in either the theta-band, alpha-band or the beta-band. We added this information to the revised manuscript (p. 6, line 124-125):

“*These effects were not observed in the spindle range (P > 0.77) or any other frequency band (P > 0.33), nor in REM sleep (S1 Fig).”*

2. *“- The methods describe performing the analyses on both EEG power and EEG energy, but the results only describe the effects for energy, and power is not discussed. Could the authors clarify what the results of their other analysis were or provide rationale for not discussing power? Power is the more commonly used metric, and it's not clear whether this is because it showed no differences, or because there was another reason to focus on the energy metric.”*

**Authors’ response**: We thank the reviewer for this important question. Given how this study represent a *first in man* investigation of genetic differences in AQP4 expression on the nocturnal sleep EEG, we did not have *a priori* knowledge of whether effects would be intensity or time related. We therefore decided to focus on slow wave energy rather than slow wave activity because it combines measures of sleep intensity and duration, and was recently described as one of the best markers of sleep propensity in humans (Skorucak et. al., *Sleep*, 2018), which is also noted in the main results (p. 5, line 120-122). This approach allowed us to further reduce the number of statistical comparisons and thereby to minimize type 1 errors. As mentioned in the method section (statistical analysis, p. 14), we only performed one overall mixed model for both power and energy before retaining to the energy measure.

However, we acknowledge that missing EEG power data may give an impression of omission. We therefore redid the analysis corresponding to Figure 2, for whole night NREM EEG slow wave activity and included the new figure as supplementary Figure 2. The results show a similar significant modulation by the *AQP4 haplotype*. We added this information to the manuscript (p. 5-6, line 122-124):

*EEG quantification revealed that the HtMi-carriers produced more SWE than the HtMa homozygotes (‘genotype’: P < 0.03; Figure 2A), an effect paralleled by post-hoc slow wave power analysis (S2 Fig).*

3. *“- The authors interpret this phenomenon as a feedback loop from glymphatics to CSF energy. That is certainly possible but it seems possible there are other explanations as well - for example, might this haplotype covary with other genetic determinants of sleep architecture?”*

**Authors’ response:** We thank the reviewer for this interesting question. The strength of hypothesis driven candidate gene studies generally is that the pathway of causality is clear. Because sleep or sleep loss cannot change our genetic variants, it must be the SNPs that affect sleep. Moreover, we are not aware of any literature suggesting that previously sleep associated SNPs should be co-expressed with AQP4. Due to the hypothesis driven nature of the data, we did test whether the Apolipoprotein E (APOE) haplotype could potentially be associated with the haplotype of AQP4, without observing any association (see suppl. Table 1). However, because the 1000 genome project database, to the best of our knowledge, does not allow us to test for possible associations between SNPs across genes and chromosomes, we cannot rule out this possibility. We therefore added this reservation to the Results and discussion section (p. 9, line 201-203) as follows:

*“Although we cannot rule out possible confounding associations between the AQP4-haplotype and other sleep regulating genes, our observations may indirectly suggest that AQP4 dependent fluid flow within the neuropil is regulated by EEG slow waves during NREM sleep.”*

4. *“- Is it correct and not a typo that sleep was scored in 20 second epochs, rather than typical 30 second epochs described in the cited reference? (this is fine for the reported analysis, but note that the reference states 30 s).”*

**Authors’ response:** We appreciate the reviewers’ eye for detail. The original scoring rules by Rechtschaffen & Kales (1968) included the broader epoch definition of 20 - 30 s, which was changed in the new scoring rules from 2007 to ensure a standard epoch length of 30 s. The original rules were more broadly defined and although they did allow for the use of frontal and occipital derivations in scoring sleep slow waves and alpha activity during wakefulness, they did not strictly require it. Because our earliest data are from around the time when the new rules were defined, we encompassed the new rules in our scoring procedure, but retained the 20 s epoch length, which for basic research provides an improved scoring resolution. To be correct in our referencing, the revised manuscript now cites the Rechtschaffen & Kales (1968) paper instead of the newer scoring rules.

5. *“- the analysis relies on the implication that the power analysis and hypothesis were designed a priori to performing genetic analyses, rather than selected post hoc. The methods section confirms that this was a priori but I'd recommend briefly adding that to the main text as well.”*

**Authors’ response:** As the reviewer points out, this indeed was an *a priori* analysis based on a clear hypothesis and targeted SNP genotyping. To make this point clearer, we modified the end of the introduction (page 4. Line 95-97) to reflect this aspect:

*“To investigate this association, a haplotype spanning AQP4 was genotyped a priori in a previously collected sample of 123 healthy participants from controlled sleep deprivation studies.”*

6. *“- add units to x-axis of fig 2.b, c”*

**Authors’ response:** We again appreciate this reviewer’s sense for detail. The unit Hertz (Hz) was indeed missing from the x-axis of Figure 2, which we have now added to the revised version of the manuscript.

**Reviewer #2:**

*“This is an interesting study investigating the AQP4 haplotypes and NREM sleep slow wave relationship. I was pleased to have the opportunity to review this work. Hypothesis is clearly stated and backed by earlier research. Study is based on analysis of data pooled from former studies and genotyping for AQP4. The topic is appropriate for this journal and manuscript is well written.*

*I have some comments on the methods that may impact the clarity of the findings”*

**Authors’ response:** We thank the Reviewer for the constructive and positive evaluation of our work.

1. *“How were the subjects recruited? Readers would benefit from a general description of who the subjects were and the basic procedure of recruitment.”*

**Authors’ response:** To fit the data to the short format communication, this description was kept to a minimum in the original version and only included as references to previously published work. However, we agree that a more formal description is beneficial. We therefore modified the Population section of the methods (p. 10, lines 237 - 240) to entail more details on the general procedure used:

*“Subjects were recruited by public advertisements seeking participants for scientific studies, or via billboard advertisements at the university buildings in Zurich. All subjects received a monetary compensation for their participation. Written and informed consent was obtained from all participants before the experiments as required according to the principles in the Declaration of Helsinki.”*

2. *“Both groups have relatively low percentage of females. Why is that? Did the authors consider making an analysis comparing the sexes?”*

**Authors’ response:** This is an important scientific question. We principally oppose scientific studies that ignore potential gender bias. As documented in the method section (p. 11), this study comprised data from six previous sleep studies. Unfortunately, because most of these were set up to run for multiple weeks and applied a randomized, cross-over design, women were excluded to minimize well-known effects from the menstrual cycle on the sleep EEG (for recent review see e.g.: Baker & Driver, Sleep Medicine, 2007). The 12 included women are all from the same study, which only ran for a single week where women were recorded at comparable times of their monthly cycle (for more details see: Bachmann et al., Sleep, 2012).

With only 12 females in the sample and a power analysis suggesting that 78 subjects are required to detect a 5% difference in EEG energy (p. 5 line 108), we do not believe that a comparison of the female participants is meaningful in the current sample. To document this, we added the following statement to the legend of the demographics table (S1 Table, p. 19, line 516-518):

*“**Given the low number of females in the six included studies, a potential interaction between AQP4-haplotype and gender could not be addressed in the current paper.”*

3*. “Although small in numbers, seven individuals homozygous for the minor allele were identified, but excluded from the analysis. I think it would be interesting for the reader to have the information about the kinds of results this small group had and a short discussion about a possible dose response effect.”*
 **Authors’ response:** While we agree with the reviewer and would be most interested to further investigate the dose-response effect of the AQP4-haplotype, we must again emphasize that our power analysis show that a much larger group size is required (>70 subjects) to adequately shed light on this question. Any investigation in the seven subjects’ homozygotes of the HtMi allele will be biased at best, and at worst, may give a false impression of the effect. In the revised version of our manuscript (p. 8-9 line 199-201) we encourage future studies designed to answer this specific question:

*“The low number of included HtMi homozygotes did not allow us to test whether the observed effects on sleep follow a dose response relationship, a question that should be addressed in future studies.”*

Moreover, we would like to highlight that no HtMi homozygotes were excluded from the study. Rather, the seven HtMi homozygotes were pooled with the HtMi heterozygotes to create our HtMi carrier group, enabling us to investigate the effect of harboring a HtMi-allele by means of dominant analysis. The description of the groups and the genetic comparison has been made clearer in the revised manuscript (p. 5, line 109–111):

*“The AQP4-haplotypes were compared by means of dominant analysis whereby 45* *heterozygous subjects and 7 homozygotes were denoted as carriers of the minor allele (HtMi) and compared to 71 individuals* *homozygous for the major allele (HtMa) variant (S1 Data)”*

**Reviewer #3, Jack A Wells:**

*“This study uncovers some interesting new evidence that links various measures of sleep quality (EEG during sleep/awake and participant survey) to genes related to AQP4 in the human brain.*

*Given emerging evidence for the interplay between AQP4, sleep, the glymphatic system and measures of neuronal activity from EEG, and the possible role of this interplay in the aetiology of neurodegenerative disease, these data are an interesting development towards better understanding these mechanisms in the human brain.  As such, I believe that the data presented in this manuscript would be of interest to the readers of Plos Biology and beyond. The data analysis and statistical approach appears appropriate to support the conclusions made, however,  I do not have first hand experience of the analysis of EEG or genetic haplotype data and therefore I cannot give detailed and informed feedback on the analytical methods employed here.*

*There are some issues in the way the data is contextualized to the current literature that I believe should be addressed prior to possible publication:”*

**Authors’ response:** We thank Dr. Jack A Wells for his thoughtful and critical feedback to our manuscripts, which we have addressed point-by-point below.

1. *“The author should avoid unnecessary speculation of possible mechanisms (specifically glymphatic activity) that may mediate or link the correlations that they have observed in their measurements:”*

**Authors’ response:** We agree that speculations should be backed up by data and worded to appropriately reflect caveats associated with the results. However, we do see it as an important role of the author to adequately reflect on and interpret results in relation to their hypothesis.

e.g:

1a.*” 'AQP4 HtMi-carriers have a stronger parenchymal CSF flow' - the authors have no evidence for this as CSF flow was not measured - the caveats to this statement should be more clearly stated.”*

**Authors’ response:** This quote is taken out of context and therefore no longer adequately reflects the statement made. The original full sentence reads (Original manuscript: p. 6, line 105-107):

The results match our initial hypothesis suggesting that in an attempt to compensate for a reduced AQP4 expression (23), AQP4 HtMi-carriers have a stronger parenchymal CSF flow and increased SWE in NREM sleep (Figure 1A).

Nevertheless, we acknowledge that this statement could be written more elegantly as done in the revised manuscript (p. 6 line 130-134) as follows:

*“The results appear in line with our initial hypothesis suggesting that to compensate for a genetic reduction in AQP4 expression (23), AQP4 HtMi-carriers increase SWE during NREM sleep,* *perhaps by an upregulation of parenchymal CSF flow (Fig 1A).”*

1b. *“'our findings support the presence of an innate glymphatic-sleep feedback loop' - again no measurements of glymphatics are made in this study. Although there is evidence in the rodent brain that links glymphatic function to AQP4 expression, changes to AQP4 may also modulate several other physiological changes in the brain such as exchange at the blood brain barrier. The wording should be toned down to acknowledge such uncertainty at the current time..”*

**Authors’ response:** We appreciate these thoughtful comments from the reviewer and agree that there are other ways by which the data can be interpreted, including modified blood brain barrier exchange. As suggested, we modified the paragraph in question (p. 7, line 166-170) as follows:

*“**The increased SWE in the low AQP4-protein expressing HtMi-carriers indicates regulatory feedback between sleep intensity and AQP4 dependent water exchange (Figure 1A). Future studies are warranted to better understand whether the increase in SWE is also associated with increased CSF clearance or perhaps indicates that sleep intensity is related to an exchange of fluids across the blood brain barrier. “*

1c.*“ 'and suggests deep NREM sleep, not AQP4, as the main regulator of glymphatic flow'  - In my opinion this is overly speculative given the lack of glymphatic measurements in this study and the current uncertainty of the relationship of glymphatic flow with sleep in the human brain. Again the wording should be adjusted to reflect the current experimental evidence.”*

**Authors’ response:** We have rewritten the paragraph in question and removed the referenced sentence (see previous answer for details).

1d. *“ 'These data suggest that alterations in AQP4-dependent parenchymal CSF flow' - again there are no measures of parenchymal CSF flow made here. I think the authors should be careful to assume that physiologically relevant differences in AQP4 expression closely relate to differences in glymphatic function in the human brain given that the existing evidence that describes this relationship comes from comparing glymphatic flow in a WT and AQP4 knockout mouse (ie. all or nothing) and from correlations in mouse models of ageing or brain pathology (e.g. TBI) where other changes to brain physiology other then AQP4 are present that could also contribute to the correlations observed.”*

**Authors’ response:** We agree that this sentence require reformulation. In the revised version of our manuscript (p. 8, line 195 - 196) we have removed the reference to parenchymal CSF flow.

*“These data suggest that alterations in AQP4-expression have cognitive consequences, unveiled during sleep deprivation.*

1e. *“ 'our data provides novel 146 evidence for the existence of sleep dependent AQP4-driven CSF pulsations' - the author should more carefully word this statement given they have no measures of CSF pulsations.. firstly it is not known how the differences in AQP4 between the genetic variants explored here may effect glymphatic function in the human brain (the changes may be so subtle as to have minimal effect compared to, vascular pulseation for example and secondly changes in AQP4 expression may affect other physiological process that related to sleep in addition to the glymphatic system (for example classical bulk CSF flow).”*

**Authors’ response:** We appreciate the careful considerations of our text by the reviewer. However, we again must oppose only citing part of our sentences. In this case, the sentence compares effects of genetic variation in AQP4 on cognitive decline in Alzheimer’s patients (Burfeind et al., Alzheimers Dement, 2017), with our data showing that comparable AQP4-variants modulate sleep intensity. We agree that future studies are needed to better understand the molecular mechanisms linking these two independent results, yet the current most logical explanation is the glymphatic pathway.

We modified the sentence to better reflect the uncertainties correctly identified by the reviewer (p. 9, line 221-227):

*“Given that SNPs associated with the HtMi variant affect the cognitive decline in Alzheimer’s patients [15], the here described modulation of sleep intensity by the AQP4-haplotype provides a* *tantalizing link between Alzheimer’s disease, sleep intensity and AQP4 in humans. Although future studies are warranted to better understand the underlying molecular mechanisms governing this effect, the data is consistent with the hypothesized impact from the glymphatic pathway. “*

2. *“In addition, the authors should avoid claiming evidence for causality when they have evidence for correlation. For example, other genetic changes that are not investigated in this work could be important for the differences in sleep behaviour that is observed.”*

**Authors’ response:** We thank the reviewer for this important point. The strength of hypothesis driven candidate gene studies generally is that the pathway of causality is clear. Because sleep or sleep loss cannot change our genetic variants, it must be the SNPs that affect sleep. Moreover, we are not aware of any literature suggesting that previously sleep associated SNPs should be co-expressed with AQP4. Due to the hypothesis driven nature of the data, we did test whether the Apolipoprotein E (APOE) haplotype could potentially be associated with the haplotype of AQP4, without observing any association (see S1 Table). However, because the 1000 genome project database, to the best of our knowledge, does not allow us to test for possible associations between SNPs across genes and chromosomes, we cannot rule out this possibility. We therefore added this reservation to the Results and discussion section (p. 9, line 201-203) as follows:

*“Although we cannot rule out possible confounding associations between the AQP4-haplotype and other sleep regulating genes, our observations may indirectly suggest that AQP4 dependent fluid flow within the neuropil is regulated by EEG slow waves during NREM sleep.”*

e.g.

2a. *“AQP4-haplotype modulates slow waves in NREM sleep*

*The title and and similar text should be appropriately toned down in acknowledgement of this.”*

**Authors’ response:** As requested by the reviewer, we toned down the title of our manuscript and adapted similar formulations throughout the manuscript as follows:

*Title:*

*“Haplotype of the astrocytic water channel AQP4 is associated with slow wave energy regulation in human NREM sleep”*

The Figure 2 title (p. 6, line 140):

*“AQP4 haplotype is associated with EEG energy regulation in the slow wave range”*

Second title in Results and discussion section (p. 5 line 115):

*“AQP4-haplotype is associated with a distinct modulation of slow waves in NREM sleep”*

Third title in Results and discussion section (p. 7 line 154-155):

*“EEG markers of sleep homeostasis in NREM sleep are associated with the AQP4-haplotype”*

The Figure 3 title (p. 7, line 174)

*“Time course of EEG slow wave production is associated with the AQP4 haplotype”*

Fourth title in Results and discussion section (p. 8 line 185-186):

*“The AQP4-haplotype is associated with a modulation of subjective and objective responses to prolonged wakefulness”*

Sentence in the Results and discussion section (Page 8. Line 197-199):

*“by showing that the AQP4-haplotype is associated with a modulation of NREM slow wave energy and the restorative effect of sleep on cognitive functions.”*

2b. *“Introduction: 'facilitates a circulation of nutrients'  - to my understanding there have been no studies to demonstrate that the glymphatic system is a physiologically-important carrier of 'nutrients' to the brain.  Could the authors clarify what 'nutrients' they are referring to and reference accordingly.”*

**Authors’ response:** We thank the reviewer for pointing out this issue. Although preliminary data suggest that such processes take place, to the best of our knowledge, these effects have not yet been published. We therefore removed this statement from the revised version of our manuscript (p.3 line 47-48).

*“, facilitates the removal of waste by generating a convective flow of CSF and interstitial fluid [1,2].”*

2c. *“ 'is generated by arterial pulsations from the heartbeat and respiration (3,4).': I can see that reference 3 (Maestre) provides evidence that arterial pulsation drives perivascular CSF movement but what is the evidence that respiration is an important mechanism? Reference 4 does not provide this as there is no measurements of glymphatic function in this work. Can the authors clarify where the evidence for respiration being a driver for glymphatic function derives from?*”

**Authors’ response:** We thank the reviewer for this interesting question. While we agree that it is still unclear whether the cardiac and respiratory pulsations observed in Kiviniemi et al., JCBFM, 2016 (original Ref. 4) represent glymphatic flow, there is some evidence in the Maestre et al., Nat Com, 2018 (original Ref. 3) that respiration may modulate glymphatic flow. This is described in the supplementary material, Figure S4 of Maestre et al., 2018. These data details that in some mice, a substantial effect from the respiration on CSF fluid flow was observed. Nevertheless, we agree with the reviewer that our statement should appropriately reflect the literature, which is why we have adapted our sentence in the revised version of our manuscript (p. 3, line 48-50) as follows:

*“Firstly, bulk flow of CSF through perivascular spaces is generated by arterial pulsations from the heartbeat and potentially to a lesser extent by pulsations from the respiration [3].”*

2d*. “ 'glymphatic flow is known to be positively correlated with slow wave production (10)' - an important caveat of this important study is the use of difference anaesthesia protocols to investigate correlations between EEG measures and glymphatic inflow. I think it is important to mention this caveat to readers who may not be aware of this..”*

**Authors’ response:** We agree that this is an important detail, which we now include in the revised version of the manuscript (p. 3, line 55-56)

*“, glymphatic flow is positively correlated with slow wave intensity during different types of anesthesia [9].”*

2e. *“ 'and recently sleep driven parenchymal CSF pulsations in the fourth ventricle were demonstrated in 56 the human brain (13), providing the first evidence for human glymphatic mechanisms'”*
*“-    Can the authors clarify what a parenchymal CSF pulsation is (and how it is measured)? -I found this hard to understand..”*

**Authors’ response:** We removed the wording ‘CSF pulsation’ from the manuscript and rewrote our reference to the Fultz et al., 2019 paper (page 3, line 59-60):

*“…, and recently sleep dependent pulsations were demonstrated in the fourth ventricle in the human brain [12].”*

4. *“-    Can the author clarify exactly how the measurements presented in ref 13 provide evidence for glymphatic mechanisms in the human brain?”*

**Authors’ response:** Although we appreciate this question, we do not believe that this short report is the appropriate place to discuss this statement, nor to the discuss the evidence presented in Fultz et al, 2019. We have therefore removed the statement about ‘glymphatic mechanisms’ (p. 3, line 60) in the revised version of our manuscript.

**Editorial comments:**

“*Based on the reviews, we will probably accept this manuscript for publication, assuming that you will modify the manuscript to address the points raised by the reviewers. Please also make sure to address the data and other policy-related requests noted at the end of this email.”*

**Authors’ response**: We thank the editorial team for their overall positive evaluation of our manuscript. As requested, we address all points raised by the editorial team and reviewers below.

1. *“Please note that PLOS does not accept references to data not shown. Thus, as pointed out by reviewers 1, please include those data either in the main body of the manuscript or in the supporting information.”*

**Authors’ response**: We have adapted the manuscript according to these requirements. All data is now presented, either in the main text or supplements and referenced accordingly.

2. *“ETHICS STATEMENT: -- Please include the ID number of your protocols approved by the ethics committee of the Canton of Zurich for research on human subjects.*

*-- Please indicate whether the written consent given by participants was informed.*

*--Please indicate if you conducted your experiments in accordance to the principles expressed in the Declaration of Helsinki or any other national or international ethical guidelines.”*

**Authors’ response**: This information was missing from the original manuscript and only included as a reference to previous publications. We acknowledge that this does not comply with *PLoS Biology* guidelines and therefore have added this information to the method section of the manuscript (10, lines 233-240) as follows:

*“Ethics statement*

*The study protocols were approved by the ethics committee of the Canton of Zurich for research on human subjects (Cantonal Ethics Committee reference numbers: E-39/2006 [29]; 2012-0398 [30]; E-24/2007 [31]; EK-Nr. 786 [32]; 2015-0424 [33]). As previously required, the oldest data set [28] was approved by local institutional review board. Subjects were recruited by public advertisements seeking participants for scientific studies, or via billboard advertisements at the university buildings in Zurich. All subjects received a monetary compensation for their participation. Written and informed consent was obtained from all participants before the experiments as required according to the principles in the Declaration of Helsinki.”*

3. “*DATA POLICY: 1) Supplementary files (e.g., excel). Please ensure that all data files are uploaded as 'Supporting Information' and are invariably referred to (in the manuscript, figure legends, and the Description field when uploading your files) using the following format verbatim: S1 Data, S2 Data, etc.*

*Regardless of the method selected, please ensure that you provide the individual numerical values that underlie the summary data displayed in the following figure panels: Figures 2A-C, 3AB (including insets), 4A-C, and S1AB.”*

**Authors’ response**: To comply with PLoS Biology guidelines we have prepared the requested raw data spreadsheets documenting the main results of the manuscripts. These are named S1 Data (genotypes), S2 Data (whole night EEG energy and power), S3 Data (cycle wise EEG energy and power), S4 Data (cognitive performance measures (SSS, PVT median speed and PVT lapses), S5 Data (REM power), and will be uploaded along with the revised manuscript. Moreover, as requested, we refer to these new data sheets in the revised manuscript as appropriate. We also updated the Data Availability statement:

*“Anonymized data relevant to this manuscript is included in supplementary datasheets (S1-S5 Data). For further information contact the Neurobiology Research Unit in Copenhagen (Dr. Holst: holst@nru.dk, or MD Ulv Larsen: sara.larsen@nru.dk) or the Psychopharmacology Laboratory at the University of Zurich (Prof. Landolt: landolt@pharma.uzh.ch).”*

Also note that to better match the figure layout to PLoS Biology, we reorganized Figure 4 from a portrait to landscape format.
